# Supplementary material for: PYGM mRNA expression in McArdle disease: Demographic, clinical, morphological and genetic features
Source: PLoS One. 2020 Jul 31;15(7):e0236597. doi: 10.1371/journal.pone.0236597 (PMC7394413; doi:10.1371/journal.pone.0236597)
Supplement: S1 Table — Legend: bp: base pair (DOCX) [file pone.0236597.s001.docx]

Supplementary table 1. Primer sequences and expected amplicons for *PYGM* and *RPL13α (internal control)*

| Gene | Primer Sequence (5´- 3`) | Amplicon size (bp) |
| --- | --- | --- |
| *PYGM* | F- TGGCCAAAGTGAAGCAGGAA  R- CGCTTCACCTGGATGTCGAA | 106 |
| *RPL13α* | F- TGAGGACCTCTGTGTATTTGTCAA  R- CCTGGAGGAGAAGAGGAAAGAGA | 126 |
