# Supplementary material for: PYGM mRNA expression in McArdle disease: Demographic, clinical, morphological and genetic features
Source: PLoS One. 2020 Jul 31;15(7):e0236597. doi: 10.1371/journal.pone.0236597 (PMC7394413; doi:10.1371/journal.pone.0236597)
Supplement: S2 Table — (DOCX) [file pone.0236597.s002.docx]

| Suplementary table 2: Brazilian McArdles patients' *PYGM* variations | | | | | | |  |
| --- | --- | --- | --- | --- | --- | --- | --- |
|  |  | predicted | dbSNP | classification | ClinVar/HGMD/ExAC | HGMD# | patients with |
| NG_013018.1 | NM_005609.3 | protein |  |  |  |  | the variant |
| g.5965C>T | c.148C>T | p.Arg50Ter | rs116987552 | pathogenic | ClinVar/HGMD | CM930629 | 8 |
| g.7469A>C | c.527A>C | p.Gln176Pro | rs747495987 | VUS | ExAC/gnomAD |  | 1 |
| g.7890G>A | c.613G>A | p.Gly205Ser | rs119103251 | pathogenic | ClinVar/ExAC |  | 1 |
| g.14119G>A | c.1827G>A | p.Lys609= | rs119103259 | pathogenic | ExAC/HGMD | CS032423 | 1 |
| g.14370C>T | c.1948C>T | p.Arg650Ter | rs114073621 | pathogenic | HGMD | CM071957 | 1 |
| g.15138C>A | c.1975C>A | p.Pro659Thr | - | VUS | - |  | 1 |
| g.15187C>T | c.2024C>T | p.Ser675Leu | rs794727189 | VUS | ClinVar |  | 1 |
| g.15286A>T | c.2123A>T | p.Asn708Ile | - | VUS | - |  | 2 |
| g.18920T>A | c.2392T>C | p.Trp798Arg | rs119103258 | pathogenic | ClinVar/ExAC |  | 1 |
| g.14126A>G | c.1827+7C>T | - | rs532747 | benign | ClinVar/ExAC |  | 2 |
