# Supplementary material for: PYGM mRNA expression in McArdle disease: Demographic, clinical, morphological and genetic features
Source: PLoS One. 2020 Jul 31;15(7):e0236597. doi: 10.1371/journal.pone.0236597 (PMC7394413; doi:10.1371/journal.pone.0236597)
Supplement: S3 Table — (DOCX) [file pone.0236597.s003.docx]

| Table 2: Association between mRNA expression and different variables. | | | | |
| --- | --- | --- | --- | --- |
| Variables | Normoexpression  N = 7 (46.7%) | Hypoexpression  N = 8 (53.3%) | Difference between values | p* |
| Ammonia T0 | 61.9 (21.2-102.7) † | 35.0 (15.2-54.9) † | 26.9 (-9.52-63.3) † | 0.133 |
| Ammonia T1 | 144.6 (22.1- 267.1) † | 186.8 (73.5-300.2) † | -42.3 ( -193.0-108.5) † | 0.552 |
| Δ Ammonia | 82.7 (20.3- 185.6) † | 151.8 (51.1- 252.5) † | -69.2 (-200.5-62.2) † | 0.273 |
| CK diag (IU/L) | 20939.9 (8178.3- 50058.0) † | 13392.0(2683.9-24100.0) † | 7547.8(-18574.5-33670.3) † | 0.543 |
| CK rest (IU/L) | 3111.9 (236.4-5987.3) † | 2639.3(65.0-5213.5) † | 472.6(-2985.7-3930.9) † | 0.772 |
| Δ CK (IU/L) | 17828.0(10893.0- 46549.0) † | 10752.8(618.0-22123.5) † | 7075.3 (-19020.3-33170.8) † | 0.568 |
| % internal nuclei | 8.7 (3.8-13.5) † | 10.1 (7.2-13.0) † | -1.44 (-6.3-3.4) † | 0.534 |
| Mean muscle fiber diameter type 1 | 65.6 (56.7-74.4) † | 54.5 (42.1-66.9) † | 11.0 (-3.1-25.2) † | 0.116 |
| Mean muscle fiber diameter type 2 | 63.8 (57.8-69.7) † | 58.2 (46.4-69.9) † | 5.6 (-6.9-18.1) † | 0.354 |
| % vacuoles | 24.3 (11.4-37.3) † | 23.6 (8.4-38.7) † | 0.79 (-17.5-19.1) † | 0.926 |
| Grip strength (kg) | 32.0 (5.3-58.7) † | 37.3 (16.6-58.1) † | -5.3 (-34.8-24.2) † | 0.701 |
| Legend: † Min and max values; * significance value p< 0.05; CK: creatine kinase; diag: diagnosis; *Student t test; T0: time zero; T1: time after 1 minute | | | | |
