# Supplementary material for: PYGM mRNA expression in McArdle disease: Demographic, clinical, morphological and genetic features
Source: PLoS One. 2020 Jul 31;15(7):e0236597. doi: 10.1371/journal.pone.0236597 (PMC7394413; doi:10.1371/journal.pone.0236597)
Supplement: Attachment — Submitted filename: Reviewer #2 comments.doc [file pone.0236597.s004.doc]

Dear Authors,

It is very interesting to read a Brazilian study in a rare disease. I would suggest a few adjustments to improve the quality of the manuscript as listed below:

Line 45: suggestion: to replace the term “cramps” by “muscle contracture”

Line 47: please correct grammar “ at rest”

Line 55: there has been research corelating sedentariness and McArdle symptoms severity – if the authors would like to quickly comment it in the intro to be more accurate

Line 63: prospective study based on case notes (for the clinical phenotype)? Why this study was considered a prospective one? Please clarify

Line 70: did the NMD symptoms of the control group overlap with McArdle disease phenotype?

Line 73 - 78: Please review English grammar of this whole paragraph – example: “Diagnosis inclusion criteria of McArdle disease”

Line 79: ICF for what? Genetic testing, or case report publication, or was this part of a research project? Perhaps it worth linking this section with line 154 (section 1.9) and keep the ICF info altogether

Please review the concept of muscle cramps x muscle contracture in McArdle

Line 96: I suppose the most important aspects of the muscle biopsy is to describe how the absence myophosphorylase was assessed, as it was one of the criteria for the diagnosis – or used to exclude a McArdle disease diagnosis in the control group. Did you assess regenerating fibers?

Line 102: what’s the grip strength assessment? Did you mean you assessed ammonia as part of the forearm exercise test?

Line 201: “None of our data was  related to PYGM expression.” If those patients have mcardle disease and the phenotype matches the genetic diagnosis, why would you state that your data is not related to PYGM? Please clarify what you mean and re-write this statement.

Line 205: please provide reference for this hypothesis

Line 239: phosphorylase stain may fade. Please confirm why these 3 patients had mcardle and not a faded staining – did you stain this biopsy with a healthy control at the same slide to assess technical issues and misdiagnosis?

Did you assess lactate? Why it was not described in table 1?

Why you did not use the hallmark of McArdle disease do confirm the phenotype? (the second wind phenomenon)

Why the lack of lactate following exercise was not reported or used as a diagnostics criteria?

In general I believe you may have a very interesting manuscript to be published, but it would be great if you could please review key concepts of GSDV, muscle biopsy staining/GSDV diagnosis and the manuscript writing.
