# Supplementary material for: PYGM mRNA expression in McArdle disease: Demographic, clinical, morphological and genetic features
Source: PLoS One. 2020 Jul 31;15(7):e0236597. doi: 10.1371/journal.pone.0236597 (PMC7394413; doi:10.1371/journal.pone.0236597)
Supplement: Attachment — Submitted filename: Letter Plos one - answer to reviewers.docx [file pone.0236597.s005.docx]

1. Comments academic editor

-There is one major absent in your article, that is to have considered this publication: Missense mutations have unexpected consequences: The McArdle disease paradigm. Hum Mutat. 2018. In this manuscript it is reported that the lack of phenotype-genotype relationship, may be explained by a mechanism controlling translation of messengers containing PYGM mutations, which was blocking protein production in mostly all patients. So, this would be the explanation of the absence of genotype-phenotype correlation.

Thank you for the reading indication. It really clarifies a bit more about the complex mechanism associated to lack of protein in McArdle disease. This information was added to discussion session:

García-Consuegra et al, 2018, evaluated the unexpected consequences of missense mutations in McArdles muscles. They observed that 95% of patients, irrespective of the PYGM genotype, had no glycogen phosphorylase in their muscles and proposed that missense mutations could likely alter PYGM mRNA secondary structure, leading to ribosome stalling and subsequent degradation. Our findings corroborate their observations.

-In relation with this publication, did you have chance to analyse muscle glycogen protein by western blot in the muscle biopsies of your patients? These results would be a high contribution, given our current knowledge of the disease.

I would like to have done the WB, but unfortunately there were no more muscle’s samples to perform it from most of patients.Considering the mRNA expression more sensitive than protein detection (WB) we chose to study the mRNA expression .

- you talk about normoexpression and hipoexpression in Table 2, but there is no mention of the criteria for this classification in the manuscript.

Answer: The description of conditions to be classified as normo or hipoexpressed was added to methods: McArdle patients were divided according to the degree of PYGM expression, compared to the average expression of this gene in the control group: "hypoexpression" was considered when *PYGM* values of 2^-DCt were below the standard deviation in the control group. Measurements within the limits of the standard deviation values in the control group were considered "normoexpression", and values of 2^-DCt of *PYGM* above the standard deviation in the the control group were considered "hyperexpression".

- In Supplementary Table 3 and 4, patients are identified with letters and in the next table with numbers.

Answer: Yes, you are right;We unified the tables and used only numbers toidentify thepatients.

- This affirmation in the abstract it is not true: “mRNA expression and its association with clinical, morphological, and genetic aspects of the disease have not been studied previously”. The mRNA expression and its association with genetic aspects of the disease have been studied previously as you mention in your paper.

Answer: I meant to say:“…clinical,morphological, and genetic aspects as a setand not “mRNA expression and its association with only genetic aspects”. It was rewritten in the text in order to be more comprehensible”.

-Table 2: this table for me does not add any important information to the manuscript, I would just remove it.

Answer: Table 2 contains the values obtained by the expression analysis, one of the objectives of the research. It showed no difference among the groups, but readers can be interested in the values obtained. It was kept as a supplementary table 3.

-Supplementary Table 3 and 4: There is information repetitive between these two tables, that should be fused in one. And this information should become not supplementary but a Table of your manuscript.

Answer: We agree. So we fused both tables and named as Table 2 in the reviewed manuscript.

-There is no sense in making a ROC curve for differentiating patients from controls, when you have so many patients which PYGM expression levels similar to the controls (I will just eliminate this form the article).

Answer: Ok, we’ve discarded it.

-In the patients in which you have not found all the PYGM mutations (there are deletions or intronic mutations no possible to detect with the techniques you have been used), you cannot say that they are normal, you can just mention mutation not found or PYGM variation identified with unknown effect. Also, in these patients, I cannot understand how you can assess the PTC number, if you have no idea of the mutations that these patients are carrying or you have just a mutation of unknown effect, you cannot have the information on the PTC number.

We do agree with you. We will classify these cases as “unknown” for number of PTCs.

- It would be very important to add to you tables the age at onset on the disease. An enormous delay in the diagnosis of this patients have been described. I would add this information in the fuse supplementary table 3 and 4.

Answer: We tried to do this but allpatients had no idea when the disease started.

They were only able to say: it started in childhood. We are sorry that is not possible to add this information precisely. We mention this fault in the results section.

- In your histological analysis you mention a fibber predominance of type 2 among the patients. However, there is not raw data on the muscle biopsy/per patient, however you can find it of fibber diameter, although no differences have been found at this level. Can you supply this information so readers can understand what is the magnitude of this difference?

We have all this information about type 1 and type 2 fibers comparison:

Quantitative variablesas the number of Type 1 and Type 2 fibers were presented by means and 95% confidence intervals, since the variables had a normal distribution (Shapiro-Wilk test, p> 0.05). To test the hypothesis that patients with McArdle have more type 2 fibers than type 1, Pearson's correlation test (r) was used. This data was added to statistical analysis session.

there was no significant difference between the percentage of Type 1 and type 2 fibers(r=-0.076).However, 80% (n=12) of the McArdle’s patientshad more type 2 muscle fibers than type 1. The mean muscle fiber diameter exhibited a normal size variation.

This information was added to results session.

However, when we compared the number of patients with type 1 and type 2 fibers in the sample, 80% (n=12) of them showed a larger number of type 2 fibers in relation to type 1 (20%, n=3). (this information was in results session). This is the reason why we did not include the raw data. On the other side, if we consider only the 12 patients with a larger number of type 2 fibers, the statistical analysis will be significant. Do you think that would be interesting to present the statistical data of the 12 cases? Please see these results:there is a moderate correlation (r between 0.5 and 0.7) but with a significant r for p <0.10 and not for p <0.05.Below, you see the raw data with 15 patients: I could include them in supplementary data.

-Also, in the discussion you should add and mention your results in relation with this article: “McArdle disease does not affect skeletal muscle fibre type profiles in humans”. Biol Open. 2014

Answer: The following information was added to the text: “To date, very few reports exist about fibre type composition muscle fibre type of in McArdle patients, specifically. Felice et al (ref) reported type 1 muscle fibre atrophy in three patients with McArdle’s disease. In contrast, Kohn et al, in an elegant study, analysed the three MHC isoforms (I, IIa and IIx) in the McArdle samples and compared them with the control ones. They did not find difference in isoform expression between the McArdle patients and healthy controls. Hereafter, new studies should investigate muscle fibre composition of McArdle patients.”

2. Reviewer #1:

-Why try to link Pygm mRNA levels to clinical phenotype without any consideration to protein levels? Most Pygm mutations cause a complete absence of my phosphorylase protein, and thus causing the disease phenotype. Besides, in the manuscript is described as an inclusion criteria "the lack of my phosphorylase staining in the muscle biopsies", this is, no my phosphorylase protein. Then, if all 15 patients had a complete absence of my phosphorylase protein, why different Pygmy mRNA levels should be related to a different clinical phenotype? Or at least, why didn't you analyse it by western blot? This is for me a major issue that should be answered and clarified. If the authors feel this reviewer has not completely understood their point, please discuss it.

Answer: Because the mRNA expression is more sensitive than protein detection(WB). So, we decided to study the mRNA expression in an attempt to clarify if the phenotypic variability could be due to differences in the expression level. I would like to have done the WB, but unfortunately there were no more muscles’ samples to perform it from most of patients. Also, all patients included in this study had performed muscle biopsy and myophosphorilase was tested by a qualitative test. It wasabsent in all.

-Abstract line 26: PYGM (glycogen phosphorylase gene)-muscle should be added (Muscle glycogen phosphorylase gene)

Answer: The information has been added to the text.

-Demographic and Clinical Data line 86: There is no necessity to describe in detail all the Martinuzzi scale grades, with the mention of reference (6) is more than enough.

Answer: Ok. The information was removed from the text.

-Pygm mRNA expression line 124: Even though it is specified that brachial biceps was used for morphology analysis, it is not specified whether the same muscle was used for mRNA analysis. Although one can imagine that the same biopsy was used for both things, it must be specified.

Answer: Yes, the same muscle was used for both, morphology and mRNA analysis. We added this information to the text.

3. Reviewer #2:

Line 45: suggestion: to replace the term “cramps” by “muscle contracture”

Answer: ok. The term was replaced.

Line 47: please correct grammar “at rest”

Answer: The word was corrected.

Line 55: there has been research correlating sedentariness and McArdle symptoms severity – if the authors would like to quickly comment it in the intro to be more accurate

Answer:Ok, I added the phrase toLine 48: Additionally, the sedentariness may aggravate the exercise intolerance by further reducing the limited oxidative capacity caused by blocked glycogenolysis.

Line 63: prospective study based on case notes (for the clinical phenotype)? Why this study was considered a prospective one? Please clarify

Answer:In the beginning of the study, we had just 9 patients without molecular diagnosis. As time went by, I was able to follow the patients, complete data and include more patients to the research.

Line 70: did the NMD symptoms of the control group overlap with McArdle disease phenotype?

Answer: No, the symptoms were diverse among controls; there was not a pattern; myalgia, increased CK levels, muscle weakness, were observed as isolated symptoms; No samples from patients with contractures was included.

Line 73 - 78: Please review English grammar of this whole paragraph – example: “Diagnosis inclusion criteria of McArdle disease”

Answer: We rewrote the paragraph as: “The diagnostic inclusion criteria of McArdle disease included typical clinical history of exercise intolerance with persistent elevated CK at rest and absence of myophosphorylase staining on muscle biopsy. [5] In addition, a positive result for MIFET (Modified Ischemic Forearm Exercise Test), characterized by flat lactate levels and an exaggerated rise of ammonia during exercise, [9] was also considered to the diagnosis. All patients showed positive PAS staining inside the subsarcolemmal vacuoles. Patients met all inclusion criteria to be part of the study”.

Line 79: ICF for what? Genetic testing, or case report publication, or was this part of a research project? Perhaps it worth linking this section with line 160 (section 1.9) and keep the ICF info altogether

Answer: Ok. We’vediscarded this information from line 79 and joinedthem in line 154, altogether. It was a project so, we needed to get the signed consent form.

Please review the concept of muscle cramps x muscle contracture in McArdle

Answer:We were in doubt which term would be better to include as most patients use the term cramps; also, there are several good articles that use the term muscle cramps (Rubio JC, Garcia-Consuegra I, Nogales-Gadea G, et al. A proposed molecular diagnostic flowchart for myophosphorylase deficiency (McArdle disease) in blood samples from Spanish patients. Hum Mutat. 2007;28(2):203‐204.;Aquaron R, Bergé-Lefranc JL, Pellissier JF, et al. Molecular characterization of myophosphorylase deficiency (McArdle disease) in 34 patients from Southern France: identification of 10 new mutations. Absence of genotype-phenotype correlation. Neuromuscul Disord. 2007;17(3):235‐241.)

However, we do agree with you that “contractures” is more adequate, as cramps come from changes in motor neuron excitability. Thus, we’ve changed the term “cramps” for “contracture”.

Line 96: I suppose the most important aspects of the muscle biopsy is to describe how the absence myophosphorylase was assessed, as it was one of the criteria for the diagnosis – or used to exclude a McArdle disease diagnosis in the control group. Did you assess regenerating fibers?

Answer: All muscle biopsies were performed and analysed by dr. Alzira. The set of histological and histochemical stains applied for all biopsies (suspect of McArdle and controls) was: H&E, Gomori, NADH, SDH, COX-SDH, PAS, ORO, phosphofructokinase.Also, the myophosphorylase stain, a qualitative histochemical test,was performed to obtain the diagnosis:the absence of stainingwas considered as negative for myophosphorylase reaction.Measurements were done according Dubowitz’s method. This information was added to the text.

No, we did not assess regenerating fibers.

Line 102:

- what’s the grip strength assessment?

Answer: The purpose of this test is to measure the maximum isometric strength of the hand and forearm muscles in order to reproduce the exercise. It is used during Modified Ischemic Forearm Exercise Test(MIFET).

-Did you mean you assessed ammonia as part of the forearm exercise test?

Answer: Yes, during the MIFET, we always assess lactate and ammonia,in order to confirm the diagnosis or exclude it.

Line 201: “None of our data was  related to PYGM expression.” If those patients have mcardle disease and the phenotype matches the genetic diagnosis, why would you state that your data is not related to PYGM? Please clarify what you mean and re-write this statement.

Answer: We meant that mRNA expression was not related to our data (clinical and laboratory data).

Line 205: please provide reference for this hypothesis

Answer:It is an author conclusion. So, it cannot be referenced.

Line 239: phosphorylase stain may fade. Please confirm why these 3 patients had mcardle and not a faded staining – did you stain this biopsy with a healthy control at the same slide to assess technical issues and misdiagnosis?

Answer: We always do the stain with a control in the same slide.

Did you assess lactate? Why it was not described in table 1?

Answer: Usually the lactate is dosed at rest and after exercise during the MIFETin order to get the diagnosis. In McArdle’s patients, at rest it isalways normaland after exercise, itshows a plateau, typical of McArdle disease. So, we think that it is unnecessary to include these values in table 1.

Why you did not use the hallmark of McArdle disease do confirm the phenotype? (the second wind phenomenon)

Answer: because the MIFET test is standardized in my university. Also, they are equivalent in terms of results.

Why the lack of lactate following exercise was not reported or used as a diagnostics criterion?

Answer: It was described at setting session in methods. Specifically at “In addition, apositive result for MIFET (Modified Ischemic Forearm Exercise Test), characterized by flat lactate levels and an exaggerated rise of ammonia during exercise, [9].”
